# Analysis of clinical features and prognostic factors in Takayasu arteritis involving pulmonary hypertension: A retrospective study

**DOI:** 10.1097/MD.0000000000037992

**Published:** 2024-05-03

**Authors:** Jinxia Wang, Kai Lei, Jinxia Li, Yanan Zhang, Shuhong Chi, Zhengping Zhang, Lingyan Huang, Xia Yang

**Affiliations:** a Ningxia Medical University, Yinchuan, China; b Department of Respiratory and Critical Care Medicine, General Hospital of Ningxia Medical University, Yinchuan, China; c Department of Rheumatology, General Hospital of Ningxia Medical University, Yinchuan, China; d Department of Radiology, General Hospital of Ningxia Medical University, Yinchuan, China; e Department of Pathology, General Hospital of Ningxia Medical University, Yinchuan, China.

**Keywords:** clinical features, prognosis, pulmonary hypertension, retrospective analysis, Takayasu arteritis

## Abstract

**Background:**

Multiple takayasu arteritis (TA) is a chronic nonspecific large to medium vasculitis disease that mainly accumulates the aorta and its branches. Pulmonary vascular disease is often seen as stenosis and occlusion, and patients may show no moderate to severe pulmonary hypertension (PH). This study aims to summarize the clinical characteristics and analysis of prognostic factors in patients with PH caused by TA.

**Methods:**

Patients diagnosed with aortitis involving the pulmonary artery by pulmonary arteriography or pulmonary artery and total aortic computed tomography arteriography (CTA). All patients underwent detailed clinical assessment, laboratory data collection, and analysis of imaging data. Patients were followed up and factors affecting the prognosis of the pulmonary arteries were analyzed.

**Results:**

Most of the patients’ complaints were chest tightness, shortness of breath, decreased activity tolerance, hemoptysis and chest pain. 56.90% of the patients were in at the time of admission. Echocardiographic estimation of pulmonary artery systolic pressure was 90.39 ± 22.87 mm Hg. In terms of laboratory tests, 39.66%% of the patients had elevated C-reactive protein and erythrocyte sedimentation rate, and amino-terminal natriuretic peptide precursor on admission. In terms of imaging, all patients had pulmonary artery involvement, which was combined with aortic involvement in 31.03%. Nuclide lung perfusion/ventilation imaging of the patients revealed multiple perfusion defects/absences in the segmental and subsegmental distribution of the lungs. Univariate Cox regression model analysis suggested that patients’ WHO functional class at admission, age ≧ 51 years at the time of consultation, and amino-terminal natriuretic peptide precursor ≧ 3500 pg/mL were factors affecting the prognosis. Further multifactorial Cox regression model analysis suggested amino-terminal natriuretic peptide precursor ≧ 3500 pg/mL was an independent predictor of poor prognosis with a hazard ratio (HR) value of 5.248.

**Conclusion:**

Electrocardiogram and echocardiogram may suggest an increased right heart load; some patients have elevated serum inflammatory indexes. Characteristic imaging manifestations include widening of the main pulmonary artery, multiple pulmonary segmental and subsegmental stenoses.

**Key Points**

- Pulmonary hypertension caused by Takayasu arteritis often shows imaging changes.
- Aortic computed tomography angiography (CTA) can improve the diagnostic rate of Takayasu arteritis.
- Amino-terminal natriuretic peptide precursor ≧ 3500 pg/mL was an independent predictor of poor prognosis.

## 1. Introduction

Takayasu arteritis (TA) is a chronic, nonspecific, large-medium total vasculitis disease primarily involving the aorta and its branches.^[1]^ The incidence of pulmonary vasculitis has been reported in the literature to vary from 10% to 50% of TA^[2]^; subsequent studies have found that the involved pulmonary arteries have similar vasculitis pathology to their body arteries.^[3]^ The majority of patients with pulmonary vasculitis have insidious clinical symptoms, often lacking in the acute phase; in the chronic phase, symptoms such as right heart failure gradually appear due to stenosis and occlusion of the pulmonary artery, and the disease is already in an advanced stage. Moreover, some clinicians do not have sufficient knowledge of pulmonary vasculitis, which greatly contributes to the delay in diagnosis and treatment of this disease.^[4,5]^

The clinical diagnosis of TA generally refers to the criteria recommended by the American Rheumatism Association in 1990^[6]^: age of onset ≦ 40 years old; intermittent claudication; diminished brachial arterial pulsation; difference in systolic blood pressure of the upper extremities of the 2 sides >10 mm Hg (1 mm Hg = 0.133 kPa); vascular murmur in the area where the subclavian artery connects to the aorta; abnormal arteriography, except for other causes such as arteriosclerosis. TA is diagnosed when 3 or more of the above criteria are present, with a sensitivity of 90.5% and a specificity of 97.8%. However, patients with pulmonary vasculitis who do not involve the rest of the aortic branches at the same time often lack the corresponding clinical symptoms and signs, so the diagnostic value of TA based on the above clinical diagnostic criteria for the diagnosis of pulmonary vasculitis is relatively limited. More comprehensive and operational diagnostic criteria are urgently needed to improve physicians’ and patients’ understanding of pulmonary vasculitis.

The latest TA staging has further updated and supplemented previous TA staging criteria^[7]^: type I, mainly involving branches of the aortic arch; type IIa, ascending aorta + aortic arch and its branches; type IIb, ascending aorta + aortic arch and its branches + descending aorta; type III, descending aorta±renal arteries; type IV, abdominal aorta±renal arteries only; and type V, IIb + IV. In addition, if the coronary or pulmonary arteries are involved, they are labeled C(+) and P(+).

This classification included pulmonary vasculitis in the classification of aortitis for the first time, however, it did not further indicate the characteristics of this type of pulmonary vasculitis.^[8]^ As mentioned above, concomitant involvement of the pulmonary arteries by TA is not uncommon, but involvement of the pulmonary arteries alone is rare, and its incidence has been reported in the literature to account for only 3% to 4% of aortitis, with only sporadic cases or small groups of cases being reported, and there is a lack of clinical case studies of pulmonary vasculitis in a large group of patients to date.^[9]^

In this study, we retrospectively analyzed the clinical data of patients with TA involving the pulmonary arteries and summarized the clinical features of the disease in order to improve the early recognition and diagnosis of the disease. We also followed up on the enrolled patients with pulmonary vasculitis and analyzed the relevant factors affecting the prognosis in order to improve the overall understanding of pulmonary vasculitis.

## 2. Methods

### 2.1. General information

The case data of 58 patients treated at the General Hospital of Ningxia Medical University from September 2009 to December 2021 were retrospectively analyzed. The diagnostic criteria were based on the 1990 American College of Rheumatology classification criteria for multiple aortitis. This research was supported by the Ethics Committee of General Hospital of Ningxia Medical University, and informed consent was obtained from all individual participants included in the study.

Inclusion criteria: age of onset < 40 years old; Meets diagnostic criteria for pulmonary arterial hypertension (PAH),^[10]^ mean pulmonary artery pressure ≥ 25 mm Hg (1 mm Hg = 0.133 kPa) and pulmonary capillary wedge pressure <15 mm Hg measured by right heart catheterization (RHC) at rest, with pulmonary vascular resistance > 3 Wood Units. The diagnosis of pulmonary vasculitis was confirmed in all patients by pulmonary arteriography or computed tomography (CTA) of the pulmonary arteries and total aorta.

Exclusion criteria: atherosclerosis, muscle fiber dysplasia and other causes.

### 2.2. Research methodology

#### 2.2.1. Assessment of baseline situation

Patients’ demographic data, medical history, physical examination findings, blood laboratory parameters, electrocardiogram, echocardiogram, pulmonary and somatic arterial angiography and imaging data such as CTA and nuclear pulmonary perfusion/ventilation imaging were collected. All patients were assessed for World Health Organization (WHO) functional classification of pulmonary hypertension (PH) on admission and some patients underwent a 6-minute walking distance test.

Six-minute walk distance test: It was standardized according to the American College of Chest Physicians 2002 guidelines for the 6-minute walk test.

### 2.3. RHC

Procedure: The RHC site is usually chosen to be the right internal jugular vein or femoral vein. The patient lies supine on the operating table in the catheterization laboratory, and is routinely disinfected and toweled, with 1% lidocaine local anesthesia. The Swan-Ganz floating catheter enters from the internal jugular vein or the femoral vein, and then passes through the superior and inferior vena cava, the right atrium, the right ventricle, the main pulmonary artery, and the right and left pulmonary arteries, and the pressures are measured in the various cardiac chambers respectively, and blood is taken to measure the oxygen saturation. When the floating catheter enters the right ventricle and fills the balloon, it readily enters the pulmonary artery and nests in the distal pulmonary artery, and small pulmonary artery wedge pressure can be measured.

Measurement indicators: height, weight, body surface area, base calorie; right atrial pressure: average pressure; right ventricular pressure: systolic/diastolic/average pressure, right ventricular end-diastolic pressure; pulmonary artery pressure: systolic/diastolic/average pressure; average pulmonary artery wedge pressure; calculation of total lung resistance and pulmonary artery resistance; body artery pressure: systolic/diastolic/average pressure; body and pulmonary circulation blood flow; fick method of calculating cardiac output, cardiac index; superior vena cava, inferior vena cava, right atrium, right ventricle and pulmonary artery segmental blood oxygen saturation; body artery oxygen saturation is mostly based on femoral artery blood; mixed venous oxygen saturation using the average of the superior vena cava and inferior vena cava oxygen saturation.

Calculation of pulmonary vascular compliance: pulmonary vascular compliance (mL/mm Hg) = right ventricular output per beat (mL)/pulmonary artery pulse pressure (mm Hg); pulmonary artery pulse pressure = pulmonary artery systolic pressure - diastolic pressure.

### 2.4. Follow-up

Follow-up each patient was followed up every 3 to 6 months by telephone, text message, or outpatient visit, with the primary follow-up outcome event being all-cause mortality. Patients were asked in detail about their symptoms and exercise tolerance at each follow-up visit.

### 2.5. Statistical analysis

Measurement information was expressed as mean earth standard deviation and intercept between minimum-maximum values; categorical information was expressed as percentages. Comparisons between groups were made using the t-test for continuous variables and the Chi-square test or Fisher exact probability method for categorical variables. Single-factor Cox regression analysis was used to screen for factors that might affect prognosis, and multifactor Cox regression analysis was used to analyze the independent predictive value of multifactorial effects on survival. The Kaplan–Meier method was used to plot the survival curves under the effect of different correlating factors. All data were analyzed by SPSS23.0, and *P* < 0.05 was considered statistically significant.

## 3. Results

### 3.1. Baseline characteristics

Patient profile: A total of 58 patients with pulmonary vasculitis were included in the study, and the profile of all patients is shown in Table 1. The average time from the onset of the earliest clinical symptoms to the definitive diagnosis of pulmonary vasculitis ranged from days to decades, with an average duration of more than 5 years. 95% of the patients were in the WHO PH functional class II to III at the time of their first visit to our hospital. The majority of patients presented with symptoms of chest tightness, shortness of breath, decreased activity tolerance, hemoptysis and chest pain at the time of their first visit to our hospital, while symptoms of the acute phase of vasculitis, such as fever, were rare, accounting for only 6.9% of patients.

**Table 1:** Baseline data of 58 patients with pulmonary vasculitis.

Clinical indicator	Patients with pulmonary vasculitis
Male/female (n)	14/44
Age at onset of symptoms (yr)	34.51 ± 9.78 [10–55]
≦20 yr (n, %)	4 (6.90)
21–30 yr (n, %)	15 (25.85)
31–40 yr (n, %)	23 (39.66)
41–50 yr (n, %)	12 (20.69)
51–60 yr (n, %)	3 (5.17)
≧61 yr (n, %)	1 (1.72)
Time from symptom onset to diagnosis (yr)	6.12 ± 6.45 [0.02–35.57]
BMI (kg/m^2^)	21.89 ± 3.35 [13.89–31.94]
WHO functional classification of pulmonary hypertension (n,%)	
I	1 (1.72)
II	22 (37.93)
III	33 (56.90)
IV	2 (3.45)
Symptoms (n,%)	
Chest tightness, shortness of breath	57 (98.28)
Decreased activity tolerance	50 (86.21)
Hemoptysis	21 (36.21)
Cough, phlegm	19 (32.76)
Chest pain	10 (17.24)
Fainting	6 (10.34)
Have a high temperature	4 (6.90)

Indicators are expressed as mean earth standard deviation or percentage.

BMI = body mass index. WHO = World Health Organization.

### 3.2. Laboratory inspection

Laboratory investigations in all patients with pulmonary vasculitis are detailed in Table 2 and mainly involved nonspecific inflammatory markers, routine blood tests, thrombosis and heart failure-related indicators. C-reactive protein and erythrocyte sedimentation rate are considered to be nonspecific inflammatory markers, and approximately 40% of patients with pulmonary vasculitis had elevated inflammatory markers. The mean amino-terminal natriuretic peptide precursor of the patients in this study was 1632.97 pg/mL on admission, which was significantly elevated in 86.21% of patients with pulmonary vasculitis, suggesting the presence of varying degrees of right heart insufficiency.

**Table 2:** Laboratory tests in patients with pulmonary vasculitis.

Clinical indicator	Patients with pulmonary vasculitis
Inflammation indicators	
C-reactive protein (mg/L)	12.87 ± 17.81 [1–109]
Elevated C-reactive protein (n, %)	23 (39.66)
Erythrocyte sedimentation rate (mm/h)	20.35 ± 25.72 [1–128]
Elevated erythrocyte sedimentation rate (n, %)	23 (39.66)
Routine blood test	
Blood platelet count (10^*^9/L)	224.06 ± 90.57 [68–565]
Platelet distribution width (%)	12.67 ± 3.24 [0–22]
Hemoglobin (g/L)	141.62 ± 22.93 [90–181]
Erythrocyte distribution width (%)	14.48 ± 1.82 [11.4–22.3]
Thrombus indicator	
D-dimer (μg/mL)	0.87 ± 2.06 [0.10–17.13]
Heart failure indicators	
Amino-terminal natriuretic peptide precursor (pg/mL)	1632.97 ± 1638.16 [30.25–7302.91]
<300 pg/mL (n, %)	8 (13.79)
300–1400 pg/mL (n, %)	27 (46.55)
>1400 pg/mL (n, %)	23 (39.66)
Other indicators	
Total bilirubin (μmol/L)	23.37 ± 16.15 [6.2–88.1]
Blood uric acid (μmol/L)	370.57 ± 125.35 [118.66–743.62]

Indicators are expressed as mean earth standard deviation and minimum-maximum intercept or percentage.

### 3.3. Electrocardiogram characteristics

In this study, 30 patients (51.7%) with pulmonary vasculitis had typical right ventricular hypertrophy on ECG at the time of their first visit to our hospital, that is, R/S in lead VI was >1, and nearly half of the patients had T-wave inversion in leads V1–3, which was suggestive of an increased right heart load. Echocardiography: all patients with pulmonary vasculitis underwent echocardiography on admission to assess right ventricular function, and the specific indices are shown in Table 3. The patients’ left ventricular structure and function were not abnormal; however, the right heart function was impaired, which was manifested by the enlargement of the right ventricle and the reduction of the systolic displacement distance of the tricuspid annulus. The systolic pressure of the pulmonary artery estimated from the tricuspid regurgitation velocity was significantly elevated, suggesting moderately severe PH.

**Table 3:** Echocardiographic features.

Clinical indicator	Numerical value
Left atrial internal diameter (mm)	30.48 ± 4.73 [20–49]
Left ventricular end-diastolic internal diameter (mm)	39.65 ± 7.26 [21–67]
Left ventricular ejection fraction (n, %)	65.59 ± 7.29 [28–80]
Right ventricular end-diastolic internal diameter (mm)	30.86 ± 8.06 [16–58]
Right ventricular end-diastolic internal diameter/left ventricular end-diastolic internal diameter > 1 (n, %)	16 (27.59)
Estimated pulmonary artery systolic pressure (mm Hg)	90.39 ± 22.87 [45–146]
Tricuspid annulus systolic displacement distance (mm)	15.48 ± 4.51 [8–20]
Pericardial effusion (n, %)	7 (12.07)

Indicators are expressed as mean earth standard deviation and minimum-maximum intercept or percentage.

### 3.4. Pulmonary arteriography/CTA features

All patients with pulmonary vasculitis in this study had pulmonary artery involvement, of which 18 cases (31.03%) had concomitant involvement of other aorta and its branches. The pulmonary angiographic/CTA features of patients with pulmonary vasculitis mainly showed stiffness, tortuosity, stenosis and dilatation of the pulmonary artery wall; among them, stenosis and dilatation coexisted in 27 cases, accounting for 46.55% of the total. The distribution of the affected pulmonary arteries is shown in Table 4. 93.10% of the patients with pulmonary vasculitis had bilateral involvement of different segments and subsegments of the pulmonary arteries, while unilateral involvement of the pulmonary arteries was more common in patients with right-sided pulmonary involvement. The main pulmonary arteries were significantly widened at the time of consultation, and 87.93% of the patients had widened ascending aortas at the same level, suggesting that the disease duration was long.

**Table 4:** Characteristics of pulmonary arteriography/CTA.

Pulmonary artery involvement	n = 58
Unilateral pulmonary artery involvement	
Right lung (n, %)	3 (5.17)
Left lung (n, %)	1 (1.72)
Bilateral pulmonary artery involvement	54 (93.10)
Involvement of all lung segments	
Right pulmonary artery (n, %)	32 (55.17)
Upper lobe of the right lung	
Tip segments (n, %)	42 (72.41)
Posterior segment (n, %)	43 (74.14)
Preceding paragraph (n, %)	40 (68.97)
Middle lobe of the right lung	
Outer segment (n, %)	43 (74.14)
Medial segment (n, %)	43 (74.14)
Lower lobe of the right lung	
Dorsal segment (n, %)	39 (67.24)
Inner basal segment (n, %)	42 (72.41)
Anterior basal segment (n, %)	43 (74.14)
Outer basal segment (n, %)	44 (75.86)
Posterior basal segment (n, %)	44 (75.86)
Left pulmonary artery	17 (29.31)
Upper lobe of the left lung	
Post apical segment (n, %)	29 (50.0)
Preceding paragraph (n, %)	31 (53.45)
Left lingual lobe of the lung	
Supraglottic segment (n, %)	36 (62.07)
Hypoglossal segment (n, %)	35 (60.34)
Lower lobe of the left lung	
Dorsal segment (n, %)	34 (58.62)
Inner anterior base (n, %)	41 (70.69)
Outer basal segment (n, %)	38 (65.52)
Posterior basal segment (n, %)	40 (68.97)
Same level	
Internal diameter of main pulmonary artery/ascending aorta	23.02 ± 0.24 [0.65–2.04]
Internal diameter of main pulmonary artery/ascending aorta > 1 mm	51 (87.93)

Indicators are expressed as mean earth standard deviation and minimum-maximum intercept or percentage.

### 3.5. Pulmonary perfusion/ventilation imaging and 6-minute walk test

A total of 32 patients with pulmonary vasculitis had their nuclear lung perfusion/ventilation imaging completed during hospitalization, which mainly showed multiple lung segments, subsegmental radiological perfusion defects/absences, and mismatched lung perfusion/ventilation imaging. Less than 5 lung segments were involved in a total of 1 patient, accounting for 3.13%; 5 to 10 lung segments were involved in a total of 4 patients, accounting for 12.50%; and the remaining 27 patients had more than 10 lung segments involved, accounting for 84.38%. A total of 20 patients with pulmonary vasculitis completed a 6-minute walking distance test during their hospital stay. The 6-minute walking distance was 402.79 ± 84.76 m; 50% of the patients walked 158 to 450 m, and 50% walked more than 450 m. The results of the 6-minute walking distance test were as follows.

### 3.6. Analysis of follow-up results

A total of 6 patients died at the end of follow-up, 1 male and 5 females. All patients were (38.75 ± 13.78) years old at the onset of symptoms, (46.18 ± 9.57) years old at the time of diagnosis, and (7.72 ± 9.18) years old at the time of symptom onset. 5 of the 6 patients (83.3%) were in WHO functional class III at the time of diagnosis, and the remaining 1 patient was in WHO functional class II. One patient (16.7%) had a history of syncope and 2 patients (33.3%) had hemoptysis. 2 of the 6 patients (16.7%) who died had received targeted therapy. In terms of laboratory tests, patients had low serum inflammatory markers and significantly elevated amino-terminal natriuretic peptide precursors reflecting the severity of the disease (Table 5). Indicators of imaging showed that both the aorta and its branches were involved in 3 (50.0%) of the patients who died; other signs were seen, such as widening of the pulmonary artery, enlargement of the right heart, and elevation of pulmonary artery pressure (Table 5). Hemodynamic investigations, only 2 of the deceased patients had undergone RHC, and the detailed hemodynamic indices are shown in Table 5. The patients had elevated pulmonary artery pressure, decreased pulmonary vascular compliance, elevated pulmonary artery pulse pressure, and a significant increase in pulmonary vascular resistance.

**Table 5:** Examination results of the 6 deceased patients.

Laboratory tests (n = 6)	
C-reactive protein (mg/L)	6.27 ± 6.73
Erythrocyte sedimentation rate (mm/h)	5.48 ± 3.92
Blood platelet count (×10^12^/L)	167.59 ± 43.26
Platelet distribution width (%)	13.31 ± 2.20
Hemoglobin (g/L)	158.25 ± 21.76
Erythrocyte distribution width (%)	14.59 ± 1.23
D-dimer (μg/mL)	0.57 ± 0.65
N-terminal natriuretic peptide precursor (pg/mL)	3051.78 ± 1938.06
Total bilirubin (μmol/L)	31.68 ± 16.04
Imaging (n = 6)	
CTA indicator	
Aortic involvement (n, %)	3 (50.0)
Total number of lung segments involved	11.12 ± 2.89
Width of main pulmonary artery (mm)	36.87 ± 4.36
Ratio of internal diameter of the main pulmonary artery/ascending aorta	1.21 ± 0.18
Echocardiographic indicators	
Left atrial internal diameter (mm)	30.72 ± 4.35
Left ventricular end-diastolic internal diameter (mm)	39.41 ± 6.68
Left ventricular ejection fraction (%)	63.02 ± 12.87
Right ventricular end-diastolic internal diameter (mm)	32.14 ± 7.46
Estimated pulmonary artery systolic pressure (mm Hg)	91.27 ± 18.68
Combined pericardial effusion (n, %)	1 (16.67)
Haemodynamic indicators (n = 2)	
Mixed venous oxygen saturation (%)	55.78 ± 8.59
Femoral artery oxygen saturation (%)	85.26 ± 3.48
Mean right atrial pressure (mm Hg)	5.48 ± 3.51
Pulmonary artery systolic pressure (mm Hg)	111.59 ± 24.06
Pulmonary artery diastolic pressure (mm Hg)	28.62 ± 7.34
Mean pulmonary artery pressure (mm Hg)	58.02 ± 13.01
Pulmonary circulation compliance (mL/mm Hg)	0.48 ± 0.13
Pulmonary pulse pressure (mm Hg)	82.87 ± 19.65
Pulmonary capillary wedge pressure (mm Hg)	8.79 ± 0.54
Heart rate (L/min·m^2^)	1.85 ± 0.18
Pulmonary vascular resistance (dyn·s·cm^-5^)	18.78 ± 5.39

Indicators are expressed as mean ± standard deviation or number of cases (percentage).

CTA = computed tomography arteriography.

### 3.7. Cox one-way analysis of factors affecting survival

Referring to the literature on the prognostic factors of aortitis, and taking into account the clinical situation, receiver operating characteristic curves were plotted for the continuous variables “age at diagnosis” and “amino-terminal natriuretic peptide precursor” using death as an incidence event, and the largest Jordon index was taken as the critical value for inclusion in the analysis of prognostic-related factors. Factor analysis. The hemodynamic data were not included in the Cox survival analysis because of the small number of hemodynamic data collected and the small number of cases of RHC in deceased patients. Table 6 shows the detailed results of the factors that may be associated with the prognosis of PA and were included in the one-way Cox regression model for analysis. As shown in the table, WHO functional class at the time of patient admission, age > 51 years at the time of consultation, and amino-terminal natriuretic peptide precursor ≧ 3500 pg/mL were prognostic factors.

**Table 6:** Cox one-way analysis of factors affecting survival.

Considerations	HR Value	95% confidence interval	*P* value
WHO Functional Classification of Pulmonary Hypertension	3.417	1.052–11.182	.04
Genders	1.152	0.315–4.148	.827
Duration of illness at time of consultation (yr)	1.048	0.977–1.124	.219
Age ≧ 51 yr at the time of consultation	4.848	1.596–14.584	.005
History of combined syncope	1.448	0.319–6.535	.621
History of hemoptysis	0.351	0.092–1.279	.116
Body mass index (BMI)	0.868	0.732–1.030	.101
C-reactive protein	0.959	0.890–1.031	.276
Erythrocyte sedimentation rate	0.915	0.827–1.012	.091
Blood platelet count	0.987	0.975–1.000	.058
Amino-terminal natriuretic peptide precursor ≧ 3500 pg/mL	4.784	1.374–16.627	.015
Total number of lung segments involved	0.874	0.761–1.000	.054
Aortic artery width	1.057	0.946–1.179	.345
Ratio of internal diameter of the main pulmonary artery/ascending aorta	1.136	0.086–14.653	.921
Aortic involvement	1.435	0.476–4.274	.518
Right ventricular end-diastolic internal diameter	1.010	0.947–1.083	.746
Estimation of pulmonary artery systolic pressure	0.999	0.985–1.014	.753
Combined pericardial effusion	0.935	0.207–4.182	.925

Indicators are expressed as mean ± standard deviation or number of cases (percentage).

CTA = computed tomography arteriography, WHO = World Health Organization.

### 3.8. Cox multifactorial regression analyses affecting survival

According to the results of Cox one-way analysis and combined with clinical experience, several factors such as WHO functional class at admission, age more than 51 years at the time of consultation, amino-terminal natriuretic peptide precursor ≧ 3500 pg/mL, and erythrocyte sedimentation rate were included in the multifactorial Cox regression model to do further analysis on survival, and as mentioned above, the RHC data were not included in the further analysis, and the specific results are shown in Table 7. The results showed that only the amino-terminal natriuretic peptide precursor ≧ 3500 pg/mL on admission was an independent predictor of poor prognosis, with an HR value of 5.248.

**Table 7:** Cox multifactorial analysis affecting survival.

Considerations	HR Value	95% confidence interval	*P* value
WHO Functional Classification of Pulmonary Hypertension	2.115	0.445–10.016	.342
Age ≧ 51 yr at the time of consultation	1.891	0.386–9.282	.430
Amino-terminal natriuretic peptide precursor ≧ 3500 pg/mL	5.248	1.316–20.926	.020
Erythrocyte sedimentation rate	0.879	0.825–1.001	.055

Indicators are expressed as 95% confidence intervals. WHO = World Health Organization.

### 3.9. Typical cases

#### 3.9.1. Case 1

**Figure 1.**
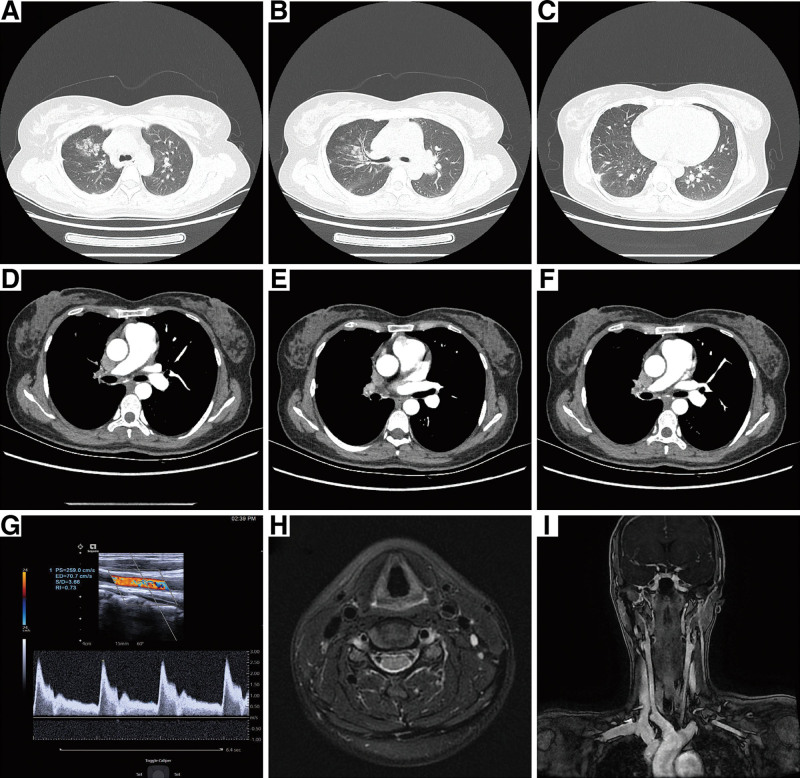
Pulmonary CT and aortic CTA. (A,B) Pulmonary CT showed ground glass exudation scattered in the right upper lung and considered the bleeding focus. (C) Subpleural cuneiform shadow. (D) Pulmonary enhanced CT showed the complete occlusion of the right pulmonary trunk and PAH. (E,F) The right bronchial artery was twisted around the right bronchus, forming a collateral circulation. (G) Color Doppler ultrasound of cervical vessels showed the diffuse and centripetal thickening of the wall and stenosis of the lumen from the beginning to the middle and distal segment of the left common carotid artery. (H,I) MRA showed the thickening of the left common carotid artery wall and a narrow lumen, and considered TA (inactive phase). CT = computed tomography, CTA = computed tomography angiography, MRA = magnetic resonance enhanced carotid angiography, PAH = pulmonary arterial hypertension.

The patient was a 30-year-old female who first attended the hospital 4 years ago complaining of dizziness and blackouts. At that time, a difference in the blood pressure of both upper limbs was observed. A carotid artery ultrasound showed that the proximal end of the left common carotid artery was uniformly thickened, and the lumen was narrowed (the narrowest stenosis rate was about 60 to 70%), and the patient was diagnosed with TA. The patient was treated with an initial dose of prednisone (50 mg/qd) and cyclophosphamide (0.4 g/every 2 weeks), and her symptoms gradually improved. The hormone was later reduced to 10 mg/qd to maintain treatment efficacy and ensure her condition remained stable. The patient experienced shortness of breath after mild activities. She had a sudden hemoptysis 9 days ago, and CTPA showed that the right pulmonary artery trunk was occluded, and the pulmonary artery was widened. The pressure of the pulmonary artery as measured by echocardiography was 53 mm Hg. After being treated with anti-infection and hemostatic drugs, a pulmonary arteriography and pulmonary artery balloon kyphoplasty were undertaken. At present, the patient has no hemoptysis (see Fig. 1).

#### 3.9.2. Case 2

**Figure 2.**
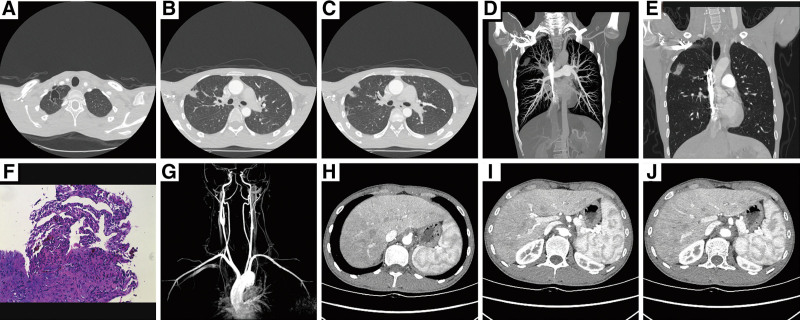
CTA and 3D reconstruction imaging results. (A) Lung CT showed cavitary lesions in the apical segment of the right upper lobe. (B,D,E) Nodular consolidation in the upper lobe of the right lung. (C) Subpleural cuneiform shadow. (F) Following a percutaneous lung puncture, the lung tissue was sent for examination and the pathology results showed coagulative necrosis, the proliferation of surrounding lung tissue, and hemosiderin deposition (a small amount of old bleeding), and the histology indicated pulmonary infarction. The pathogenic test results were negative (Periodic Acid-Schiff stain, ×40). Following CTA of the thoracic aorta, ventral aorta, iliac artery, femoral artery, popliteal artery, tibial and fibular artery a diagnosis of (active phase) TA was considered. The brachiocephalic trunk (G), bilateral subclavian arteries (G), abdominal aorta and celiac trunk (H), superior mesenteric artery (I), and involved bilateral renal arteries (J). CTA = computed tomography angiography, CT = computed tomography, TA = Takayasu arteritis.

The patient was a 23-year-old male who had suffered from dizziness and left neck pain for 2 years, accompanied by a cough and shortness of breath. Pulmonary computed tomography (CT) showed cavitary lesions in the tip of the right upper lobe and subpleural nodular consolidation lesions in the right upper lobe; the patient had previously been diagnosed with cavitary pulmonary tuberculosis at a local hospital and treated with anti-tuberculosis drugs for 9 months. The patient did not experience obvious symptom relief after therapy, and suffered from a repeated low fever and fatigue, and his left neck pain did not improve. A CTA examination suggested that his bilateral common carotid arteries, bilateral subclavian arteries, abdominal aorta, celiac trunk, superior mesenteric artery and bilateral renal arteries were thickened, and his lumen was narrowed. His ESR was 82 mm/h and interleukin (IL)-6 was 41.59 pg/mL. A diagnosis of active TA was considered. CT showed lesions in the right upper lung nodules. His etiological detection and bacteria test results for concentrated bacteria and fungi were all negative, and there was no basis for infection. The tumor markers were also negative. The puncture biopsy results suggested pulmonary infarction lesions. A diagnosis of TA lung involvement was considered, and the patient was treated with a hormone (methylprednisolone 40 mg/qd) combined with cyclophosphamide (CTX 0.4 g/every 2 weeks) and Toczumab (once a month), and his symptoms were gradually relieved. The hormone was then gradually reduced and maintained (prednisone 10 mg/qd), and the disease did not recur (see Fig. 2).

#### 3.9.3. Case 3

**Figure 3.**
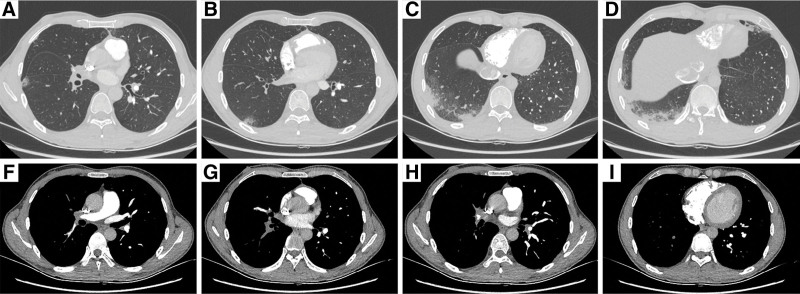
CT and CTPA imaging results of the lung. (A–D) The pulmonary infarction focus. (E) Wall thickening and lumen stenosis of the main right pulmonary artery. (F–H) Right middle lobe and lower lobe pulmonary artery branch occlusion. CT = computed tomography, CTPA = CT pulmonary angiogram.

The patient was a-25-year-old healthy male without symptoms, such as fever, fatigue, chest tightness, or shortness of breath. He suffered chest pain with hemoptysis after working for 6 days, and was diagnosed with an acute pulmonary embolism at a local hospital and given an anticoagulant treatment with low molecular weight heparin. Due to his poor response, he was transferred to a superior hospital, which found that the distal end of the right pulmonary artery trunk was thin, the right upper lobe and lower lobe basal segment arteries were not visible, the right middle lobe and lower lobe dorsal segment arteries were thickened, the lumen was narrowed, and there were multiple pulmonary infarcts under the pleura of the right lung. Echocardiography showed mild tricuspid regurgitation, PAH, and a pulmonary artery pressure of 60 mm Hg. Positron emission tomography (PET)-CT showed that the Fludeoxyglucose metabolism in the walls of the thoracic aorta and pulmonary artery was increased, and a diagnosis of TA was considered. This patient had TA with PAI as the first manifestation (see Fig. 3).

**Figure 4.**
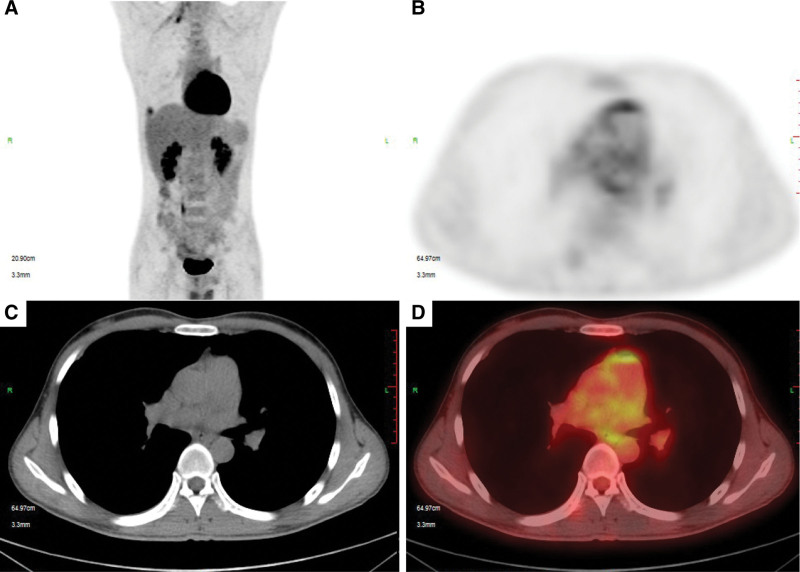
PET-CT imaging results of a patient with TA. PET-CT = positron emission tomography-computed tomography, TA = Takayasu arteritis.

The positron emission tomography-computed tomography showed that the Fludeoxyglucose metabolism in the walls of the large vessels was slightly increased, such as the thoracic aorta and pulmonary artery in the mediastinum (see Fig. 4).

## 4. Discussion

TA is a relatively common primary, immunological, chronic inflammatory arterial disease, mainly manifested as multiple, non-septic, occlusive inflammation of the aorta and its main branches, which can cause stenosis or occlusion of different parts of the vessels, resulting in dilatation of the arteries at the lesion, and in a minority of cases, aneurysm can be formed.^[11]^ The lesion vessels are most frequently involved in the cephalic and brachial arteries, and pulmonary artery involvement is rare; because some patients with pulmonary artery involvement lack clinical manifestations related to pulmonary artery involvement for a long time, it is not easy to attract the attention of clinicians, and it is easy to miss the diagnosis at an early stage, therefore, the descriptions of pulmonary vasculitis in the literature are mostly from the reports of the cases or the small-group case-observational studies.^[12]^ The patients with pulmonary vasculitis in this study took an average of more than 5 years from the first appearance of symptoms to the diagnosis of pulmonary vasculitis in our hospital, and most of the patients were accompanied by moderate or severe PH at the time of consultation, which made treatment tricky. Early diagnosis and early treatment are important for delaying disease progression and improving patient survival. Similar to the demographic characteristics of TA, pulmonary vasculitis is most common in women of childbearing age between 20 and 40 years, but the age of onset is not always limited to below 40 years.^[13]^ In the present study, 27.59% of patients were older than 40 years at the time of first symptom onset, and 6.9% of them were even older than 50 years, which shows that age older than 40 years is not a basis for ruling out pulmonary vasculitis.

The clinical symptoms of patients with pulmonary vasculitis mainly include systemic symptoms and clinical manifestations related to the affected pulmonary arteries, which can theoretically go through 3 stages: the nonspecific inflammatory stage, the vasculitic inflammatory stage and the quiescent stage.^[14]^ The nonspecific inflammatory phase mainly manifests systemic symptoms such as fever, arthralgia, and malaise, and the latter 2 phases are ischemic symptoms and signs caused by stenosis or occlusion of the pulmonary arteries.^[15]^ Systemic symptoms such as fever during the course of the patients with pulmonary vasculitis in this study were uncommon and perhaps not taken seriously by the patients due to the insidious nature of the disease. Most of the patients presented with symptoms such as exertional chest tightness and shortness of breath, decreased activity tolerance, cough, chest pain, and hemoptysis, which is consistent with the literature; 56.90% of the patients were in the WHO Pulmonary Hypertension Class III at the time of diagnosis, which indicates that the majority of the patients were in the middle to late stages of the disease, and the optimal period of treatment might have been missed, which would have a serious impact on the prognosis of the patients.

Electrocardiography and echocardiography are of little value in the etiological diagnosis of patients with pulmonary vasculitis, but they are of great significance in the assessment of right heart morphology and right heart function. In our study, more than half of the patients had significant right ventricular hypertrophy on the ECG at the time of admission to the hospital, and T-wave inversion in the right thoracic lead also suggested an increased right heart load. These electrocardiographic findings were highly consistent with the echocardiographic findings, which showed an enlarged right ventricle, a reduced systolic displacement distance of the tricuspid valve, and a combination of moderate and severe pulmonary arteries as suggested by the estimation of tricuspid regurgitation velocities. Recently, some scholars have also used echocardiography to assess the wall thickness and degree of stenosis of the affected pulmonary arteries in patients with pulmonary vasculitis, which further improves the value of echocardiography in the early identification of pulmonary vasculitis and in the assessment of its function.

Elevated erythrocyte sedimentation rate, C-reactive protein and other inflammatory markers are currently considered to be important indicators of active vascular inflammation. However, it has been shown that in 39.66% of patients with active vascular inflammation, the erythrocyte sedimentation rate was normal, suggesting that a normal erythrocyte sedimentation rate does not mean that the inflammation in the vessel wall has subsided and the lesion has stopped. The National Institutes of Health (NIH) index for assessing disease activity remains the more widely adopted standard for assessing TA lesion activity,^[16,17]^ including recurrence or exacerbation of systemic symptoms, such as fever and unexplained musculoskeletal symptoms; increased erythrocyte sedimentation rate; and new symptoms of vascular ischemia or inflammatory activity in the corresponding organ. Signs and symptoms of ischemia or inflammatory activity, such as pulselessness, vascular murmurs and claudication; and angiographic demonstration of worsening vascular lesions. If 2 or more of the above 4 items are satisfied at the same time, it suggests lesion activity. In this study, erythrocyte sedimentation rate and C-reactive protein were elevated in about 40% of the patients, while the remaining 60% were normal, suggesting that the diagnostic value of inflammatory markers for pulmonary vasculitis is also very limited, but they can provide a certain degree of guidance for determining the degree of disease activity; other inflammatory markers, such as the matrix metalloproteinase family, ortho-pentameric protein-3, and the mean platelet volume can be a favorable supplement to the assessment of TA activity. Other inflammatory markers such as matrix metalloproteinase family, n-pentaglobin-3, and mean platelet volume can be a favorable addition to the assessment of TA activity. It should be noted that since the location and extent of vascular involvement in patients with pulmonary vasculitis are different from those in patients with other types of TA, whether the above inflammatory markers can also play an indicative role in this group of patients needs to be confirmed by further studies.

In patients with TA, in the acute phase, the pathological manifestations of the disease can be seen in the infiltration of many kinds of multiple nucleated cells such as lymphocytes, plasma cells, and histiocytes, which form granulomatous changes in the intima and plasma layer; in the chronic phase, intimal fibrotic tissue hyperplasia, necrosis of the intermediate layer, and fibrosis visible in the plasma layer can be seen, which leads to lumen narrowing and occlusion; at the same time, the destruction of the whole layer of the vessel wall results in diffuse vasodilatation, aneurysm, and in situ thrombus formation.^[18,19]^ The imaging findings in this group of cases showed that involvement of the pulmonary arteries was most common in lobar and segmental arteries, followed by subpulmonary segments and their distal branches. In addition, bilateral pulmonary arteries were more frequently involved than unilateral ones, and in patients with unilateral pulmonary artery involvement, the right side was more frequently involved than the left side, which was consistent with the literature. However, coexisting stenotic and dilated lesions are not uncommon in this group of patients with pulmonary vasculitis. As mentioned above, it is not uncommon for TA to be associated with both aortic branch and pulmonary artery involvement, but it is uncommon for the pulmonary artery to be involved alone, suggesting that the pulmonary artery involvement in TA may have 2 different manifestations. In the former case, the body arteries and pulmonary arteries have the same pathological changes; in the latter case, the lesions are confined to the pulmonary arteries and often involve lung segments with larger lumen diameters. The diagnosis of TA involves a number of criteria, among which the morphology of the affected vessels, that is, the characteristic imaging changes, is the most intuitive and clear, and has the greatest value in the diagnosis of TA.

Studies have shown that the long-term prognosis of patients with TA is generally good, with Ohigashi et al^[20]^ reporting a mortality rate of 4.2% in 106 patients with TA who started before 1999 and 0% in those who started after 2000. The time from symptom onset to definitive diagnosis was shorter in patients with TA starting after 2000, and the incidence of occlusion of the aorta and its major branches and complications of moderate and severe aortic regurgitation were significantly reduced. In patients with TA starting after 2000, the time from symptom onset to definitive diagnosis, the incidence of occlusion of the aorta and its major branches, and the complications of moderate to severe aortic regurgitation have also been significantly reduced, probably due to the aggressive use of hormones and immunosuppressive drugs. Two peaks of death in patients with TA have been observed in the previous literature, in the first year after diagnosis and in the 10 to 15 years.^[21]^ The Cox survival curves of the patients in this study also suggest that the cumulative survival decline in patients with PA is uneven, with a more concentrated decline in survival within the 1st year and 3 to 5 years after diagnosis, and changes in survival beyond 10 years need to be clarified by further extended follow-up observations.

Elevated ESR has long been considered an indicator of TA vasculitis activity.^[3,22]^ Some studies have found that an ESR of <20 mm/h can indicate a poor prognosis for TA, probably because those with an elevated ESR are more likely to be diagnosed as having active lesions and actively given hormonal and immunosuppressive treatments; on the contrary, those with a normal ESR tend to be ignored by clinicians or even delayed in diagnosis and treatment. The present study also found that none of the deceased patients had an elevated ESR at the time of their first admission. However, the diagnosis of pulmonary vasculitis was clearly established in all patients in this group, and there was no delay in diagnosis and treatment in the deceased group. This may be due to the fact that the patients’ vascular disease had become chronic, and the chronic stenosis and occlusion of blood vessels led to reduced perfusion of organs, especially ventilation/perfusion mismatch caused by reduced perfusion to the lungs, which further led to a series of complications such as hypoxia in other organs and right heart failure, and the progression of the disease did not end because of the termination of the vasculitis activity. Cox unifactorial and multifactorial regression analyses showed that the ESR could not indicate the prognosis of the patients with pulmonary vasculitis, indicating that the patients with pulmonary vasculitis had no prognosis. Cox single-factor and multifactorial regression analysis found that ESR could not suggest the prognosis of patients with pulmonary vasculitis, suggesting that vasculitis activity may have a suggestive role in the diagnosis of pulmonary vasculitis, but it is not a direct factor affecting the prognosis of pulmonary vasculitis.

The patients with pulmonary vasculitis in this group were characterized by a significant increase in pulmonary artery pressure and pulmonary vascular resistance, while the pulmonary capillary wedge pressure was normal, which was classified as pulmonary precapillary PAH. Various indicators reflecting the functional status of the right ventricle such as right ventricular weight, volume, and right atrial pressure are important factors affecting the prognosis of PAH, so clinical attention should be paid to the changes in right heart function. There are many indicators that can indicate the functional status of the heart, including clinical symptoms and signs, such as whether the jugular vein is angry, peripheral swelling, syncope, etc; commonly used clinical indicators also include the WHO PH functional class, 6-minute walking distance, NT-pmBNP/BNP, systolic tricuspid annular displacement distance, and whether there is a combination of pericardial effusion, etc. In this study, the patients with PA who died in the follow-up of the malefactor were relatively older at the time of diagnosis, and the WHO functional class at the time of their first hospital admission was not as high as it was at the time of their first hospital admission. WHO functional class at admission was III-IV in most of them, and NT-proBNP was significantly elevated, which was suggested to be associated with prognosis by Cox univariate analysis. However, further Cox multifactorial regression analysis suggested that only NT-proBNP > 3500 pg/mL was an independent predictor of poor prognosis in patients with PA. It is possible that the number of patients in this group was too small, and the number of clinical events in the follow-up was too few to obtain statistically significant results. If the number of cases can be further increased, or the follow-up period can be extended, we may be able to get closer to the real-world conclusion.

This study has some limitations, firstly, the malefactor is a single center retrospective analytical study, which may be subject to selective bias. Secondly, the small number of cases included in this study affects the extension of the conclusions drawn. However, pulmonary vasculitis itself is a rare disease, and the number of cases in a single center is inevitably small. In the future, it is necessary to carry out prospective, multicentre clinical studies, and all patients with suspected aortitis should be subjected to angiography or CTA of the body arteries and pulmonary arteries, and closely followed up, in order to have a better understanding of the clinical regression of the disease and its prognosis, and thus to further promote the improvement of the diagnosis and treatment of pulmonary vasculitis.

## 5. Conclusions

Pulmonary vasculitis is most common in young women of childbearing age, and its clinical manifestations mainly include chest tightness, shortness of breath, decreased activity tolerance, chest pain, and hemoptysis. Electrocardiogram and echocardiogram may suggest increased right heart load; some patients have elevated serum inflammatory markers. Characteristic imaging findings include widening of the main pulmonary arteries, bilateral pulmonary arterial stenosis, subsegmental stenosis, occlusion, and in some patients, dilatation. In patients with pulmonary vasculitis, age >51 years at first admission, amino-terminal natriuretic peptide precursor greater than 3500 pg/mL, and high WHO functional class of PH were associated with poor prognosis, with amino-terminal natriuretic peptide precursor greater than 3500 pg/mL as an independent predictor of poor prognosis.

## Author contributions

**Conceptualization:** Yanan Zhang.

**Formal analysis:** Jinxia Wang, Jinxia Li, Shuhong Chi, Zhengping Zhang, Lingyan Huang, Xia Yang.

**Writing – original draft:** Kai Lei.

**Writing – review & editing:** Yanan Zhang.
